# Protective effect of leptin on induced apoptosis with trichostatin A on buffalo oocytes

**Published:** 2016-06-15

**Authors:** Hamid Reza Shafiei Sheykhani, Rooz Ali Batavani, Gholam Reza Najafi

**Affiliations:** 1 *Department of Theriogenology, Faculty of Veterinary Medicine, Urmia University, Urmia, Iran; *; 2 *Department of Basic Sciences, Faculty of Veterinary Medicine, Urmia University, Urmia, Iran.*

**Keywords:** Apoptosis, Buffalo, Leptin, Oocyte, Trichostatin A

## Abstract

Leptin, the 16-kDa product of the obese (ob) gene, primarily secreted from adipose tissue, has been implicated to play an important role in the regulation of food intake and energy expenditure. This study investigated protective effect of leptin on trichostatin A-induced apoptotic on *in vitro* maturation ratio of buffalo oocytes. Ovaries were collected from abattoir and were transported immediately to the laboratory by a thermos flask containing sterile normal saline with antibiotics. Oocytes were aspirated from 2 to 8 mm visible follicles. Oocytes were placed in a culture plate and then incubated at 38.5 ˚C with 5% CO_2_ in air for 24 hr. The maturation of oocytes was evaluated under a stereomicroscope. The FITC-Annexin V and propidium iodide staining method was used to detect oocyte apoptosis. In leptin treated groups with 0, 10, 50 and 100 ng mL^-1^ and groups that apoptosis was induced, the percentage of oocytes maturation was 77.03, 86.12, 85.08, and 79.89% and 59.96, 56.93 and 51.98, respectively, while the percentage of apoptosis was 8.83, 7.90, 8.58, and 9.39%, and 10.37, 11.57 and 12.03, respectively. Our findings showed that addition of 10 and 50 ng mL^-1^ leptin to IVM medium of buffalo oocytes could increase oocyte nuclear maturation, and could decrease oocyte apoptosis when trichostatin A added for inducing apoptosis.

## Introduction

Buffalo is an important livestock resource in many Asian and Mediterranean countries.^1^ In comparison with cattle, reproductive technologies have been poorly developed for buffalo. This may be due to its eproductive physiology characteristics such as late maturity, silent oestrus, distinct seasonal reproductive pattern and long calving interval,^2^^,^^3^ the low oocyte maturation rate (69.50% to 72.30%),^3^ poor oocyte recovery rate, lack of standardization for technical factors in *in vitro* embryo production (IVEP) and low *in vitro *fertilization (IVF) performance of buffalo bull spermatozoa in this species.^4^^,^^5^

If the growth of oocytes in follicle can be well managed, immature oocytes in the ovaries can be used as sources of mature oocytes for the purpose of *in vitro* fertilization program and as a source of recipient cytoplasm in somatic cell nuclear transfer to produce stem cell. Research by Ryan *et al*. showed that leptin has no direct effect on the spontaneous maturation due to inability to release the inhibition of oocyte maturation by phosphodiesterase-3B (PDE3B) inhibitor, inhibitor3-isobutyl-1-methylxanthine and milrinone, in both the denuded oocytes (DO) and cumulus oocyte complex (COC).^9^ However, a research shows that leptin significantly increased the proportion of oocytes in reaching metaphase II (MII). It proves that leptin plays a role in the maturation of nucleus and cytoplasm.^6^

Leptin, the 16-kDa product of the obese (ob) gene, has been implicated to play an important role in the regulation of food intake and energy expenditure.^7^ In addition, leptin is known to regulate diverse reproductive functions.^8^ The ob/ob mice expressing a truncated form of leptin were obese and infertile.^6^ Exogenous leptin supplementation can restore normal weight and fertility in ob/ob mice, suggesting that leptin influences reproduction in a direct way.^9^ Leptin receptor has been detected in granulosa cells, cumulus cells and oocytes in human, mouse, rat, rabbit, porcine, ewe and bovine. The presence of leptin receptor in oocyte and embryo, bovine, porcine and rabbit has been suggested that both oocytes and pre implantation embryos could react to leptin.^10^

In the last decade, the functional role of a leptin hormone-supplemented medium during *in vitro *maturation has been addressed in species such as mice,^11^ pigs,^12^ cattle,^13^ and horses.^14^ Studies in adult cattle have shown that leptin supplementation during the *in vitro *maturation of oocytes increases the proportion of oocytes reaching the blastocyst stage and blastocyst cell numbers.^13^ It seems that leptin enhances oocyte maturation by mitogen activated protein kinase (MAPK) pathway phosphorylation.^15^

Exposure of oocytes to physiological concentrations of leptin has been found to increase phosphorylation of the signal transducer and activator of transcription-3 and MAPK,^10^ decreasing levels of cAMP which promotes germinal vesicle breakdown output leading to the maturation of oocytes.^10^ Leptin reduces the proportion of apoptotic cumulus cells.^10^ An increase in the extent of apoptosis may alter connectivity between the cells of the cumulus-oocyte subsequently reducing the quality of oocytes, and the degree of apoptosis has been correlated with the developmental competence of bovine cumulus-oocyte complexes.^16^

Researches in and around apoptosis, the programmed cell death, have increased substantially since the early 1990s. It has been proved that treatment with exogenous leptin in ob/ob mice and human with congenital defect in leptin production can reintegrate the immune response and can reduce thymus atrophy with an increase in cellularity.^9^ Leptin administration in rat reduce incidence of oocyte apoptosis *in vivo*.^10^ Furthermore, it has been demonstrated that in leptin deficient mice, folliculogenesis is impaired and the apoptosis of granulosa cells is increased.^9^

The aim of this study was to investigate the protective effect of different leptin doses (10, 50, 100 ng mL^-1^) on trichostatin A-induced apoptotic on *in vitro* maturation ratio of buffalo oocytes.

## Materials and Methods

All chemicals and reagents were purchased from Sigma-Aldrich (St. Louis, USA), unless otherwise stated. Plastic dishes and six-well plates were obtained from Petes Co. (Colorado, USA). 

**Collection of oocytes. **Buffalo ovaries were collected from Urmia Abattoir, Urmia, Iran (37˚ 33΄ N, 45˚ 4΄ E). Ovaries were collected in normal saline containing antibiotics (penicillin 400 IU and streptomycin 400 mg mL^-1^) maintained at 35 to 37 ˚C and brought to laboratory within 2 hr following sampling. Prior to oocytes aspiration, extra tissues from the ovaries were removed and ovaries were washed several times in normal saline supplemented with 50 μg mL^-1^ gentamycin. Oocytes were aspirated from 2 to 8 mm visible follicles of the ovaries using an 18G hypodermic needle attached to a 10 mL disposable plastic syringe containing aspiration medium including tissue culture medium (TCM-199) fortified with 10% fetal bovine serum (FBS; Invitrogen, Carlsbad, USA)^10^ and 50 μg mL^-1 ^gentamycin.^17^ For assessment of oocytes quality, the classification of Yadav *et al*. was used,^18^ and oocytes were graded by morphological appearance of the cumulus cells investments and homogeneity of ooplasm under a zoom stereomicroscope (110×) as follow: Ι. A grade: cumulus oocyte complex (COC) with four or more layers of compact cumulus cells surrounding the zona pellucida (ZP) with evenly granulated cytoplasm, ΙΙ. B grade: COC with 1-3 layers of compact cumulus cells surrounding the ZP with evenly granulated cytoplasm, ΙΙΙ. C grade: oocyte with fibrous (expanded) cumulus layers surrounding the ZP, and ΙV. D grade: oocyte without cumulus cells and an irregular ooplasm. Only grades A and B oocytes were employed for *in vitro *maturation (IVM).

The collected oocytes were washed two times in fresh pre-warmed 4-(2-Hydroxyethyl)-1-piperazine ethane sulfonic acid (HEPES) buffered Tyrode’s medium (TL-HEPES) followed by two washings in culture medium containing TCM-199 supplemented with 10% FBS, and were then subjected to a final wash with IVM medium before transferring to the drops.

*In vitro *maturation medium included TCM-199, 10% FBS, 22 μg mL^-1^ sodium pyruvate, 0.5 IU mL^-1^ ovine follicle-stimulating hormone (oFSH), 0.5 IU mL^-1^ ovine luteinizing hormone (oLH), 1 μg mL^-1^ oestradiol, 50 μg mL^-1^ gentamycin, leptin (mouse recombinant leptin) including 0 (control), 10, 50, and 100 ng mL^-1^ and trichostatin A (100 nM).^19^^,^^20^ Good quality buffalo oocytes (batches of 10 oocytes) were placed in a culture plate containing six droplets of 50 µL of maturation medium, covered with sterilized mineral oil, and then incubated at 38.5˚C with 5% CO_2_ in air for 24 hr. Oocytes maturation was evaluated under a stereomicro-scope by detecting the first polar body extrusion which is the indicator of oocyte attaining the metaphase II stage.^18^

Cells were stained with Annexin-V, a phospholipid- binding protein that detects translocation of phosphatidyl-serine to the outer cytoplasmic membrane, an event which takes place during the early stages of apoptosis. Annexin-V-FLUOS binds in a Ca^2+^ dependent manner to negatively charged phospholipid surfaces and shows high specificity to phosphatidylserine, therefore, it stains apoptotic as well as necrotic cells. Simultaneously, cells were stained with propidium iodide (PI), a membrane impermeable stain, to distinguish between live cells and dead cells. The PI can only enter the cell when the cytoplasmic membrane has lost its integrity. Specific binding of FITC-annexin V along staining with PI was performed with an apoptosis detection kit (BD Pharmingen™, San Diego, USA) according to the manufacturer’s instructions. Half of each group were selected oocytes. Oocytes were diluted in 200 µL ABB buffer and were located gently on the siliconized slides. Afterward, 10 µL Annexin–V was added to them. The samples were incubated at room temperature in the darkness for 20 min. Then, 1 μg mL^-1^ PI was added to the samples and apoptotic oocytes were immediately detected under a fluorescence microscope (Nikon, Tokyo, Japan). After Annexin-V staining, oocytes were classified into three groups: (1) Annexin-V negative: no signal in the ooplasmic membrane; (2) Annexin-V positive (apoptotic): a clear green signal observed in the oocyte membrane; (3) Necrotic: PI positive – red nucleus indicative of membrane damage and green signal inside of oocyte.

In Annexin-V staining, the membranes containing phosphatidylinositol were bound to fluorescent dye due to inversion of oocytes membrane and apoptosis, so under the fluorescence microscope was detectable as a green staining. Non apoptotic oocytes were not stained.

**Statistical analysis. **Data on maturation and apoptosis were analyzed using SPSS (Version 19; SPSS Inc., Chicago, USA). Statistical mean and standard deviation of the mean were calculated for each group and were compared by one-way analysis of variance (ANOVA). Duncan’s test was used for the multiple comparison and least significant difference (LSD) values were calculated for significant difference between control group and treatment groups. Differences were considered significant when *p *≤ 0.05.

## Results

**Fig. 1:**
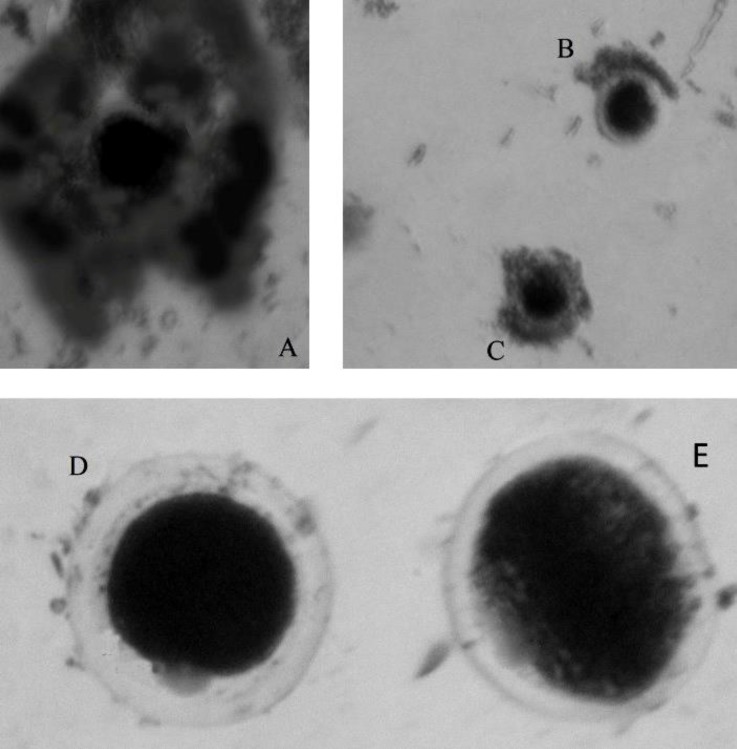
Buffalo oocytes: **A)** Oocyte after extraction from ovary, **B)** poor quality oocyte for IVM, **C)** good quality oocyte for IVM, **D)** Oocytes MII with polar body and **E)** oocyte without MII, (125×).

**Fig. 2:**
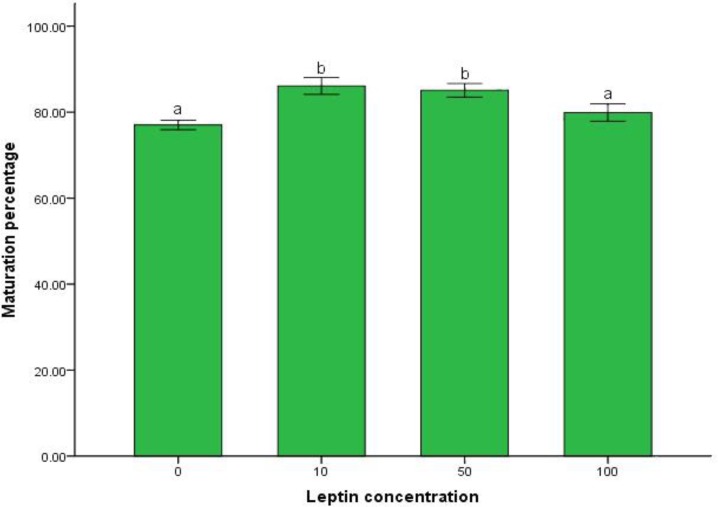
Effect of different leptin concentrations on oocyte maturation.

^ab ^Different letters indicate significant differences among the groups (*p *< 0.05).

Only oocytes enclosed with compact cumulus cells were used for maturation. Oocytes maturation was evaluated by detecting the first polar body (Fig. 1). The percentage of oocyte maturation in control group and leptin treated groups is mentioned in Figure 2. Addition of 10 and 50 ng mL^-1^ leptin to buffalo IVM medium significantly increased oocyte maturation, (*p *< 0.05). 

**Fig. 3:**
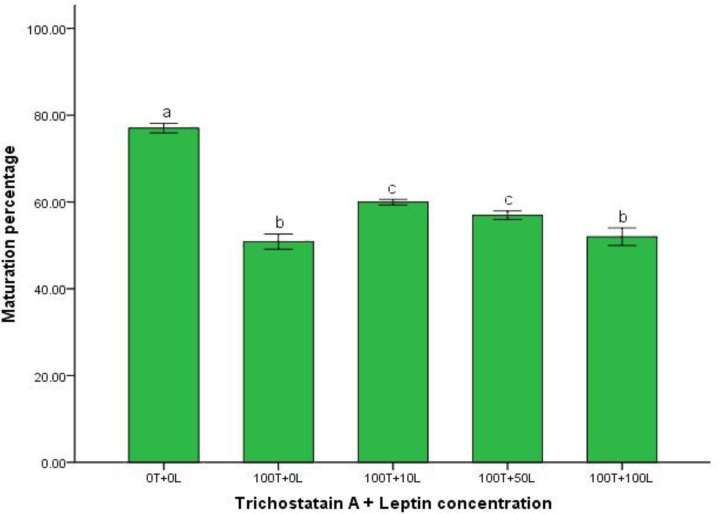
Protective effect of leptin on maturation of trichostatin A - induced apoptotic oocytes.

The percentage of oocyte maturation in control group and leptin treated groups that received trichostatin A for inducing apoptosis is mentioned in Figure 3. Addition of 10 and 50 ng mL^-1^ leptin to buffalo IVM medium that received 100 nM trichostatin A for inducing apoptosis significantly increased oocyte maturation, (*p *< 0.05).

**Fig. 4:**
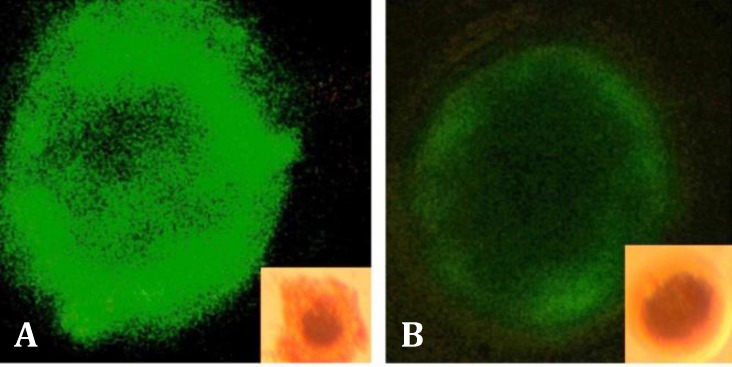
Anexin-V staining for detecting oocyte apoptosis (250×); **A)** apoptotic oocyte and **B)** non-apoptotic oocyte

**Fig. 5:**
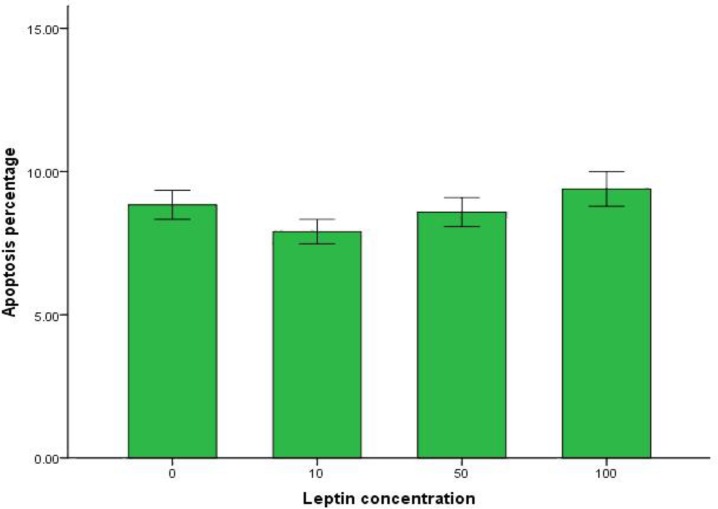
Effect of different leptin concentrations on oocyte apoptosis**.** There is no significant difference among the groups (*p *> 0.05

Annexin V-FITC and PI staining method was used to detect oocyte apoptosis (Fig. 4). The percentage of oocyte apoptosis in control group and leptin treated groups is mentioned in Figure 5. There was no significant difference in buffalo oocytes apoptosis between control group and the other leptin treated groups (*p *> 0.05).

**Fig. 6:**
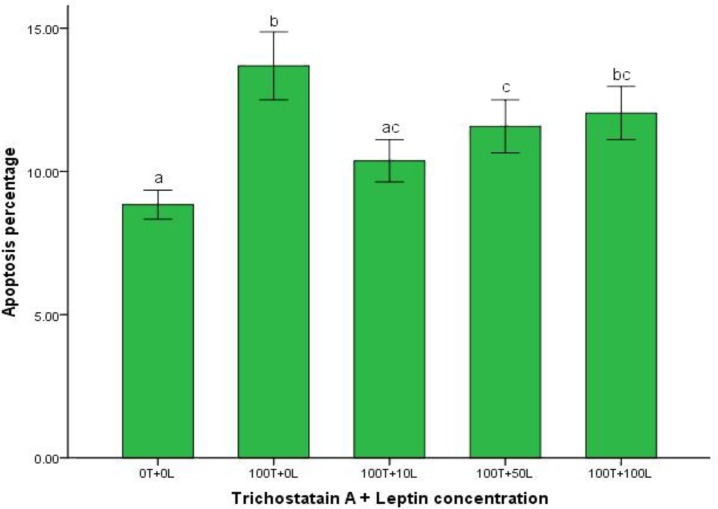
Protective effect of leptin on apoptosis of trichostatin A - induced apoptotic oocytes

^abc^ Different letters indicate significant differences among the groups (*p *< 0.05).

The percentage of oocyte apoptosis in control group and leptin treated groups that received trichostatin A for inducing apoptosis is mentioned in Figure 6. Addition of 10 and 50 ng mL^-1^ leptin to buffalo IVM medium that received 100 nM trichostatin A for inducing apoptosis significantly decreased oocyte apoptosis, (*p *< 0.05).

## Discussion

The present study was carried out to investigate the protective effect of leptin on trichostatin A-induced apoptotic on *in vitro* maturation ratio of buffalo oocytes.

Arias-Alvarez *et al*. showed that addition of leptin to IVM medium at physiological dose (10 ng mL^-1^) improves both meiotic and cytoplasmic maturation of rabbit oocytes, whereas an excessive leptin concentration does not have the extra beneficial effect.^22^ It has been established that the addition of leptin at physiological concentrations (~ 10 ng mL^-1^) enhances ability of *in vitro *maturation of adult bovine oocytes.^10^^,^^13^^,^^23^ Craig *et al*. observed that 10 ng mL^-1^ leptin during pig oocytes maturation significantly caused higher oocyte maturation rates.^15^ Moreover, in horses, Lange Consiglio *et al*. demonstrated that the addition of leptin in the range between 10 and 1000 ng mL^-1^ increases the maturation rate of equine oocytes, although the statistical significance was observed only at the concentration of 100 ng mL^-1^.^15^ These results are in agreement with our observation in buffalo which demonstrated that addition of 10 ng mL^-1^ leptin to buffalo oocyte IVM medium improves oocyte maturation.

Lu *et al*. studied the effect of leptin on *in vitro *development of buffalo embryos, showing that supplementation of 10 and 100 ng mL^-1^ leptin to *in vitro *culture medium of buffalo embryos enhanced blastocyst development in buffalo.^1^ The optimal concentration of leptin in their procedures was 10 ng mL^-1^ and they did not add leptin to IVM medium.

Histone acetylation plays an important role in the meiotic maturation of oocytes.^24^^-^^26^ This role of histone acetylation has also been shown in pig oocytes.^25^^,^^27^ The pro apoptotic effect of histone deacetylation (HDAC) inhibitors in somatic cells is described.^28^^,^^29^ A similar effect on apoptosis in somatic cells has also been seen in trichostatin A.^30^ The trichostatin A originally developed as an antifungal agent, is one of potent HDAC inhibitors, which are known to cause growth arrest and apoptosis induction of transformed cells, including urinary bladder, breast, prostate, ovary, and colon cancers. With this background, we studied trichostatin A as causing apoptosis in buffalo oocytes and protective effect of leptin on trichostatin A-induced apoptotic on *in vitro* maturation ratio of buffalo oocytes. The different effect of inhibitors of HDAC on somatic cells and aged pig oocytes could be caused by different pathways that trigger apoptosis in oocytes and somatic cells. There are studies reported that leptin exerts anti-apoptotic activity in T cells, monocytes, neuroblastoma cells, neutrophils, hippo-campal neurons and murine dendritic cells, while inducing apoptosis in human bone marrow stromal cells.^31^

Our finding showed that leptin had no significant effect on oocyte apoptosis after IVM in comparison with that in control group. Whereas, 10 and 50 ng mL^-1^ leptin had significant effect on oocyte apoptosis after IVM compared to that in control group when trichostatin A-induced apoptosis. trichostatin A inhibited the growth of HeLa cells via Bcl-2-mediated apoptosis.^32^However, there is an *in vivo *study which reported that leptin administration in rats can rescue oocytes and follicles from atresia by attenuation of apoptosis.^10^ Furthermore, leptin deficiency in mice is associated with suppression of ovarian folliculogenesis and with an increase in ovarian granulosa cell apoptosis.^33^ Leptin supplementation during bovine oocyte maturation reduces the proportion of terminal deoxynucleotidyl transferase dUTP nick end labeling (TUNEL)-positive cells per blastocyst.^13^ It has been shown that physiological doses of leptin during maturation of oocyte cumulus complex increase expression of baculoviral inhibitor of apoptosis protein repeat- containing 4 (BIRC4) mRNA transcripts, while decrease the cumulus cells apoptosis and show no beneficial effect on bovine oocyte maturation.^34^

Furthermore, Paula-Lopes *et al*. which studied the *in vitro* effect of leptin on nuclear maturation of bovine oocyte reported that leptin reduces apoptosis of cumulus cells, however, have no effect on oocyte apoptosis.^23^ Similarly, Jin *et al*. showed that addition of leptin during IVM of porcine oocytes had no effect on apoptotic cells in blastocysts.^12^ It has been demonstrated that leptin has no effect on expression of apoptotic genes in bovine blasto-cyst *in vitro*.^21^ Furthermore, Cordova *et al*. acclaimed that leptin not only has no effect on oocyte apoptosis, but also high leptin concentration increases oocyte apoptosis during IVM of prepubertal calf oocytes.^35^

In conclusion, our findings showed that addition of 10 and 50 ng mL^-1^ leptin to IVM medium of buffalo oocytes could increase oocyte nuclear maturation, and could decrease oocyte apoptosis when trichostatin A was added for inducing apoptosis. We recommend adding this hormone to IVM medium for improving oocyte maturation of this merit mammal. 
